# Increasing Versatility of the DNA Vaccines through Modification of the Subcellular Location of Plasmid-Encoded Antigen Expression in the *In Vivo* Transfected Cells

**DOI:** 10.1371/journal.pone.0077426

**Published:** 2013-10-09

**Authors:** Alicia Martinez-Lopez, Pablo García-Valtanen, María del Mar Ortega-Villaizan, Verónica Chico, Regla María Medina-Gali, Luis Perez, Julio Coll, Amparo Estepa

**Affiliations:** 1 IBMC, Miguel Hernández University, Elche, Spain; 2 INIA-SIGT–Biotecnología, Madrid, Spain; 3 LABIOFAM Pharmaceutical Laboratories, La Habana, Cuba; Instituto Butantan, Brazil

## Abstract

The route of administration of DNA vaccines can play a key role in the magnitude and quality of the immune response triggered after their administration. DNA vaccines containing the gene of the membrane-anchored glycoprotein (gpG) of the fish rhabdoviruses infectious haematopoietic necrosis virus (IHNV) or viral haematopoietic septicaemia virus (VHSV), perhaps the most effective DNA vaccines generated so far, confer maximum protection when injected intramuscularly in contrast to their low efficacy when injected intraperitoneally. In this work, taking as a model the DNA vaccine against VHSV, we focused on developing a more versatile DNA vaccine capable of inducing protective immunity regardless of the administration route used. For that, we designed two alternative constructs to gpG_1-507_ (the wild type membrane-anchored gpG of VHSV) encoding either a soluble (gpG_1-462_) or a secreted soluble (gpG_LmPle20-462_) form of the VHSV-gpG. *In vivo* immunisation/challenge assays showed that only gpG_LmPle20-462_ (the secreted soluble form) conferred protective immunity against VHSV lethal challenge via both intramuscular and intraperitoneal injection, being this the first description of a fish viral DNA vaccine that confers protection when administered intraperitoneally. Moreover, this new DNA vaccine construct also conferred protection when administered in the presence of an oil adjuvant suggesting that DNA vaccines against rhabdoviruses could be included in the formulation of current multicomponent-intaperitoneally injectable fish vaccines formulated with an oil adjuvant. On the other hand, a strong recruitment of membrane immunoglobulin expressing B cells, mainly membrane IgT, as well as *t-bet* expressing T cells, at early times post-immunisation, was specifically observed in the fish immunised with the secreted soluble form of the VHSV-gpG protein; this may indicate that the subcellular location of plasmid-encoded antigen expression in the *in vivo* transfected cells could be an important factor in determining the ways in which DNA vaccines prime the immune response.

## Introduction

DNA vaccines, one of the most notable tools developed in the field of vaccinology [1], are the simplest embodiment of vaccines since instead of administering the antigen itself, provide the gene/s that encode them [2] coded by a plasmid DNA vector. Thus, it is expected that due to their simplicity as well as their cost- and time-efficient development, they will be rapidly transferred into the pharmaceutical development pipeline [1]. In fact, four DNA vaccine products have been recently approved in the area of veterinary medicine [3]. However, DNA vaccines still face several challenges that concern their safety, antigen immunogenicity, delivery and stability, that impair their worldwide licencing.

Early in the development of the DNA vaccines it became clear that those vaccines require an appropriate delivery technology since the route of administration can play a key role in the magnitude and quality of the triggered-immune response [3,4]. This is the case of the licensed DNA vaccine against the infectious haematopoietic necrosis virus (IHNV), a fish rhabdovirus (Apex-IHN, Aqua Health Ltd, Canada) [5,6] which, when administered by intramuscular injection (i.m.) induces protective immunity in farmed salmons (protection of 85-98% of DNA-immunised fish) but only partial protection if administered by intraperitoneal injection (i.p.) [7,8].

This DNA vaccine against IHNV consists of a plasmid vector encoding the cDNA sequence of the surface antigen of the virus, the membrane-anchored glycoprotein G (gpG), under the transcriptional control of the cytomegalovirus (CMV) promoter. To date, the reason for the low efficacy of this vaccine when i.p.-delivered remains unclear. Nonetheless, it has been proposed that the transfection of antigen presenting cells (APC) or somatic cells does not occur as efficiently in the fish peritoneal cavity as it does in muscle cells [7]. In this case, a lower antigen availability to induce an optimum immune response might be the underlying cause of the i.p. vaccine failure. For that reason, studies aiming to increase the availability and diffusion of the antigen at the DNA vaccine injection site should be conducted in order to solve this problem.

In order to do so, we removed the amino acid C-terminal hydrophobic anchor (transmembrane) and the cytoplasmic domains of the VHSV-gpG, from aa 462 to 507 (of a total length of 507 aa) to obtain a soluble form of the gpG of VHSV. In addition, a soluble secreted form of the protein was obtained by replacing the N-terminal VHSV-gpG signal peptide (from aa 1 to 20) with that of the flatfish pleurocidin. This is a naturally secreted antimicrobial peptide which confers a highly secretion activity [9,10]. VHSV DNA vaccine was chosen because of the important economic losses that this virus causes to the European salmonid fish farm industry [11].

The results obtained from fish genetic immunisation assays showed that the DNA vaccine encoding the secreted soluble form of VHSV-gpG was able to protect fish against VHSV lethal challenge by using either i.p. or i.m delivery. This is the first description of a fish viral DNA vaccine that succeeded in conferring protection when administered intraperitoneally. Furthermore, this optimised DNA vaccine has a potential use as part of the multivalent vaccine formulations currently used in salmonid fish farms in light of the protection levels obtained when administered intraperitoneally as an oil-emulsion. Fish vaccines are usually administered by intraperitoneal rout. Therefore, the fact that a DNA vaccine is able to maintain its properties and its ability of protection when administered intraperitoneally and together with oil adjuvants, gives the possibility to combine it with the current vaccines using a single point of administration, simplifying the fish immunisation task.

On the other hand, our results indicate that the subcellular location of plasmid-encoded antigen expression in the *in vivo* transfected cells could be an important factor regulating the immunogenicity of DNA vaccines. In fact, the differences in the priming of the immune response by the membrane-anchored and secreted antigen were observed soon after immunisation.

## Materials and Methods

### Plasmid constructs designs

**Figure 1:**
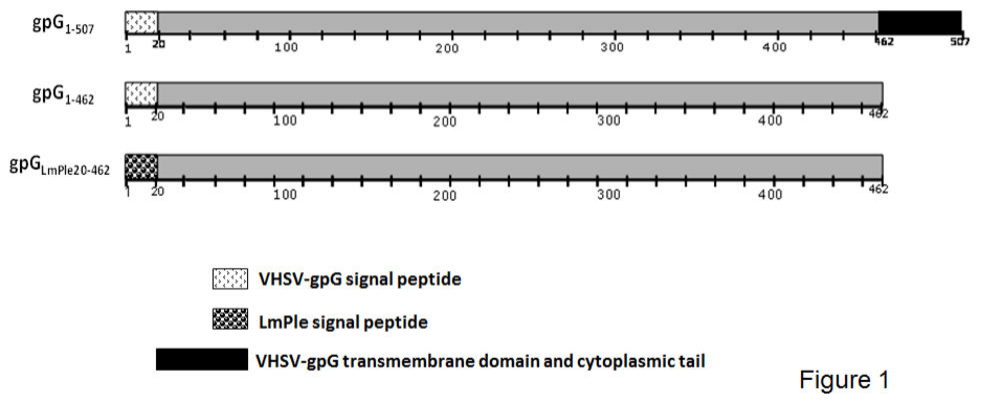
Translation of proteins corresponding to the plasmid constructs used for the experiments. VHSV-gpG constructs are shown with the amino terminus at the left. Amino acids residues from 1 to 20, VHSV-gpG signal peptide; from 20 to 462, VHSV- gpG ectodomain and from 462 to 507 VHSV-gpG transmembrane domain and cytoplasmatic tail. gpG_1-507_, whole sequence of VHSV-gpG. gpG_1-462_ comprises the VHSV-gpG ectodomain including the signal peptide sequence. gpG_LmPle20-462_ contains the VHSV- pG ectodomain and the *Limanda limanda* Pleurocidin (LmPle) signal peptide sequence.

The plasmid pAE6-gpG_1-507_ [12] encoding the wild type form of glycoprotein G (gpG_1-507_) of VHSV (Figure 1) under the control of the 5’ regulatory sequences of the carp β-actin gene was used in this work.

Using the whole sequence of VHSV-gpG_1-507_, we designed the following VHSV-gpG forms i) a soluble VHSV-gpG (the VHSV-gpG_1-462_ that lacks the transmembrane domain and cytoplasmic tail (Figure 1)) and ii) a secreted soluble VHSV-gpG, the gpG_LmPle20-462_ (Figure 1), similar to the soluble form but bearing the signal peptide of the antimicrobial peptide pleurocidin from *Limanda limanda* (gene bank accession number DQ248966).

Both, the gpG_1-462_ and gpG_LmPle20-462_ DNA sequences of VHSV-gpG were synthesised by Biost (Montreal, Canada). Each of these synthetic nucleotide sequences were then cloned into pAE6 plasmid [10] following standard procedures to yield pAE6-gpG_1-462_ and pAE6-gpG_LmPle20-462_, respectively.

### Cell culture and virus

The fish cell line EPC (Epithelioma papulosum cyprinid from *Pimephales promelas*) [13,14] purchased from the American Type Culture Collection (ATCC number CRL-2872) was used in this work.

EPC cells were maintained at 28 °C in a 5% CO_2_ atmosphere with RPMI-1640 Dutch modified (Gibco, Invitrogen corporation, UK) cell culture medium containing 10% fetal calf serum (FCS) (Sigma, Che. Co, St. Louis, Ms, USA), 1 mM Pyruvate (Gibco, Invitrogen corporation, UK), 2 mM Glutamine (Gibco), 50 µg/ml gentamicin (Gibco) and 2 µg/ml fungizone.

Viral haemorrhagic septicaemia virus strain 07.71 (VHSV_07.71_) isolated in France from rainbow trout (*Oncorhynchus mykiss*) [15] was propagated in EPC cells at 14 °C as previously reported [16]. Supernatants from VHSV_07.71_-infected EPC cell monolayers were clarified by centrifugation at 4000 x g during 30 min and kept in aliquots at -80 °C. Clarified supernatants were used for the experiments. The virus stock was titrated in 96-well plates using a previously developed immunostaining focus assay (focus forming units, f.f.u.) [17,18].

### Generation of stably-transfected EPC cell cultures expressing the VHSV-gpG forms.

EPC cell lines enriched in cells stably expressing the different VHSV-gpG forms were obtained as previously described [19], with minor modifications. Briefly, EPC cell monolayers, grown in six-well plates were co-transfected with 1.5 µg of pAE6-gpG_1-507_, pAE6-gpG_1-462_ or pAE6-gpG_LmPle20-462_ plasmid construct plus 0.25 µg of pAE6-*pac* (puromycin resistance gene) plasmid construct and incubated for 48 h at 20 °C. Puromycin resistant cells were then selected by adding 20 µg/ml of puromycin (Sigma) to the cell culture media. After 3 days the remaining puromycin-resistant cells were seeded in 96-well plates at different densities and grown in conditioned cell culture medium from non-transfected EPC cells. Then, the different density cell cultures were screened for the presence of VHSV-gpG expressing cells by real time PCR as below indicated using specific primers (Table 1), located in the central region of the VHSV G gene and therefore recognising all the three constructs. After 2 weeks, puromycin-resistant cells were transferred to 48-well plates and grown in conditioned medium and puromycin. The EPC cell cultures EPC-gpG_1-502_, EPC-gpG_1-462_ and EPC-gpG_LmPle20-462_, were grown in 6-well plates and gradually transferred into cell culture flasks. The different EPC-gpG cell lines were maintained at a rate of one pass/week in the presence of 10 µg/ml of puromycin at 20°C for over 1 year.

**Table 1:** Primer and Probe Sequences.

**Gene**	**F: Forward Primer (5’→;3’)**	**R: Reverse primer (5’→3’)**	**Probe (5’→3’)**	**ACC number/Reference**
**tcr**	AGCACCCAGACTGCCAAGCT	GAGGAGCCCTGGAACTCCA	TCT TCA TCG CTA AGA GTA CCT TCT ATG GCC TGG T	EU072699
**cd4**	GTGTGGAGGTGCTACAGGTTTTTTC	ATCGTCACCCGCTGTCTGTG		Wang, T et al. 2012
**cd8**	GAC TGC TGG CTG TGG CTT CC	CCC CGG AGC TGC CAT TCT		AF178055.1|AF178055
**ifnγ**	CAAACTGAAAGTCCACTATAAGATCTCCA	TCCTGAATTTTCCCCTTGACATATTT		Wang, T et al. 2012
**tbet**	GGTAACATGCCAGGGAACAGGA	TGGTCTATTTTTAGCTGGGTGATGTCTG		Wang, T et al. 2012
**gata3**	CCAAAAACAAGGTCATGTTCAGAAGG	TGGTGAGAGGTCGGTTGATATTGTG		Wang, T et al. 2012
**pax5**	ACGGAGATCGGATGTTCCTCTG	GATGCCGCGCTGTAGTAGTAC		Zwollo P, et al. 2008
**igm**	AAAGCCTACAAGAGGGAGACCGAT	AGAGTTATGAGGAAGAGTATGATGAAGGTG	CTCGTGTTGACTGACTGTCCATGCAGCAAC	Liang et al. 2006
**igt**	TTTTCACATGCGCCGTCAAG	AGCGAAGCCCGCCTCAG	TGT GTC GAA GTC CAC GGC GAA CAT CC	AY870265.1
**ef1α**	ACCCTCCTCTTGGTCGTTTC	TGATGACACCAACAGCAACA	GCTGTGCGTGACATGAGGCA	Raida M. K, et al. 2008
**gVHSV**	CGACCAGCTCAACTCAGGTGTCCTCAT	GGTGACTCGATAAGTCACTCTGTGCAC		AY546616

### Analysis of the expression and subcellular location of VHSV-gpG forms expressed in the stably-transfected EPC cell cultures

The expression of VHSV-gpG forms in the stably transfected EPC cells were analysed at both transcriptional and protein levels by quantitative real time RT-PCR (RT-qPCR) and immunofluorescence (IF).

For RT-qPCR assays, cell RNA extraction and cDNA synthesis were performed as described below. Quantitative PCR was carried out using primers specifically designed to detect the three forms of VHSV-gpG (Table 1). The internal reference to normalize data was the cellular 18S rRNA gene.

To detect the subcellular location of the different constructs of the VHSV-gpG expressed in the permanently transformed EPC cell cultures, cells were grown in 96-well plates and fixed with 4% paraformaldehyde (15 min at room temperature) and left untreated or permeabilised with 0,02% of Triton X100 in PBS. Then, the cells were incubated with a cocktail of anti-gpG MAbs [20], diluted 200-fold for 2 h at room temperature. After a wash with PBS, 100 μl/well of fluorescein-labelled rabbit anti-mouse IgG Ab (Sigma) diluted 200-fold was added and incubated for 45 min. To visualize nuclei cells were incubated with 1 µg/ml of the DNA stain DAPI (Sigma) for 10 min. Stained cells were viewed and photographed with an inverted fluorescence microscope (Nikon Eclipse TE2000-U; Nikon Instruments, Inc., NY) provided with a digital camera (Nikon DS-1QM).

### Detection of VHSV-gpG in the supernatants of the stably transfected EPC cell lines by HPLC

The supernatants from the different stably transfected EPC cell lines, grown in 25 cm^2^ flasks were harvested 5 days after seeding. Likewise, the supernatants from EPC cells infected as before indicated [18] were collected at day 5 post infection. The supernatants were then frozen and lyophilised. The lyophilised supernatants from EPC-gpG _1-507_, EPC-gpG_1-462_ were suspended in H_2_O. Samples from EPC-gpG _LmPle20-462_ and VHSV infected EPC cells were suspended in 50% H_2_O and 50% acetonitrile (ACN), due to solubility problems. All samples were submitted to HPLC (High Pressure Liquid Chromatography). The samples were loaded onto a C18 analytical column at 0.5ml/min to separate the molecules according to their hydrophobicity, with a linear gradient of 0% of solvent A (0.12% trifluoroacetic acid, TFA, and water) to 60% of solvent B (0.10% TFA in acetonitrile, [ACN]).

### Fusion assays

Both EPC and EPC cell lines enriched in cells stably expressing the different VHSV-gpG forms were grown on 96 well plates as above indicated. After 48 h, the cell culture medium was removed, the cells washed and the fusion triggered by incubating the cells with fusion medium [17] at pH 6 for 30 min at 14 °C. The monolayers were then washed and incubated with fusion medium at pH 7.5 for 2 h at room temperature. To evaluate the fusion, cells were fixed with cold methanol, dried and stained with Giemsa (5 mg/ml in PBS) [21]. Cells were viewed and photographed with an inverted fluorescence microscope (Nikon) provided with a digital camera (Nikon DS-1QM). As fusion positive control, EPC cells infected with VHSV (10^3^ ffu/ml) were used [18].

### Fish

Rainbow trout (*Oncorhynchus mykiss*) of 3-4 cm obtained from a VHSV-free commercial farm (Lillogen, Leon, Spain) were maintained in 50 L tanks at the University Miguel Hernandez (UMH) facilities at 12-14 °C with a re-circulating dechlorinated-water system and fed daily with a commercial diet (Trout, Leon, Spain). Prior to experiments, fish were acclimatised to laboratory conditions for 2 weeks. Animals were handled in accordance with the National and European guidelines and regulations on laboratory animal care. Animal work was approved by the UMH corresponding Ethic Committee (authorization IBM-AE-001-11).

### DNA immunisation protocol

DNA immunisation experiments were carried out following procedures previously described [12]. Briefly, fish were anaesthetized by immersion in 50 µg/ml buffered tricaine (MS-222; Sigma) prior to handling and then divided into the corresponding groups. Two independent immunisation trials were performed as follows,

First trial: to test the protection conferred by the indicated DNA vaccines, groups of 30 fish (Table 2) were intraperitoneally (i.p.) or intramuscularly injected (i.m.) with: 50 µl of PBS (non-immunised or control trout) or 50 µl of PBS containing 2 µg of the pAE6, pAE6-gpG_1-502_, pAE6-gpG_1-462_ or pAE6-gpG_LmPle20-462_ constructs. Then, cells from the fish peritoneal cavity were collected at 3 days post-immunisation. For that, the following procedure was performed: first, the mucus on the abdominal side of the fish was cleaned with ethanol; secondly, to avoid contaminating the peritoneal cells with blood, fish were exsanguinated through the dorsal aorta; third, using a 25 gauge needle, 300 µl of PBS was injected i.p. in the ventral midline. The abdominal area was then massaged for about 30 s to disperse the peritoneal cells in the injected PBS. Then, the intraperitoneal PBS containing suspended cells was collected with the same syringe and placed in RNA lysis buffer until processed.

**Table 2:** Plasmids injected intraperitonealy (i.p.) and intramuscularly (i.m.) into laboratory-reared rainbow trout.

	**FIRST TRIAL**		**SECOND TRIAL**	
Delivery route	i.m.	i.p.	i.m.	i.p.
	PBS	PBS		PBS
	Empty Plasmid	Empty Plasmid	pAE6-G_1-507_	Empty Plasmid
Plasmid	pAE6-G_1-507_	pAE6-G_1-507_		Empty Plasmid+ Oil adjuvant
	pAE6-G_1-462_	pAE6-G_1-462_		PBS + Oil adjuvant
	pAE6-G_LmPle20-462_	pAE6-G_LmPle20-462_		pAE6-G_LmPle20-462_
				pAE6-G_LmPle20-462_+ Oil adjuvant

Second trial: to test the application of conventional vaccine adjuvants along with the DNA vaccine encoding the secreted soluble form of VHSV-gpG, groups of 30 fish (Table 2) were i.p.-injected with: 50 µl of PBS, 50 µl of PBS containing 2 µg of pAE6 or 2 µg of the pAE6-gpG_LmPle20-462_ in the presence or absence of the non-mineral oil adjuvant Montanide ISA 763 AVG (Seppic, France) at a ratio of 30:70, respectively. To obtain a stable fluid emulsion in the presence of Montanide the recommendations of the manufacturer were followed. As a control of the experiment, fish were i.m-immunised with pAE6-gpG_1-502_.

In both trials, at day 30 post-immunisation blood from the caudal vein was extracted (four fish per group) to study the antibody production and neutralising activity.

### RNA isolation and cDNA synthesis

The E.Z.N.A. ® Total RNA Kit (Omega Bio-Tek, Inc., United States) was used for total RNA extraction following manufacturer’s instructions. Isolated RNAs was stored at -80 °C until used. One microgram of RNA, as estimated by a NanoDrop spectrophotometer 200 c (Thermo Fisher Scientific Inc.) was used to obtain the cDNA using M-MLV reverse transcriptase (Invitrogen) as previously described [18].

### Quantitative PCR assays

Quantitative PCR (qPCR) was performed using the ABI PRISM 7300 System (Applied Biosystems, NJ, USA) and SYBR green staining as previously described. To detect the forms of VHSV-gpG in the stably transfected EPC cell cultures or in the cells from fish intraperitoneal cavity, specific primers were designed (Table 1). Expression results were analyzed by 2^-ΔCt^ method [22] where ΔCt is determined by subtracting the 18S rRNA gene Ct value used as an endogenous control (Applied Biosystems) from the target Ct.

In the case of the *in vivo* gene expression, the primers and probes used are listed in Table 1. Gene expression results were analysed by means of the 2^−ΔΔCt^ method (Livak, K.J., 2001), using as an endogenous control for quantification the cellular elongation factor 1 alpha (EF-α) gene. cDNA from intraperitoneal-cavity cells of control fish (non-immunised fish) served as calibrator, and fold increases were calculated relative to the level of these fish. Reactions were performed in a 20-µl volume comprising 2 µl of cDNA reaction mixture, 900 nM each primer, 200 nM probe, and 10 µl of TaqMan universal PCR master mix (Applied Biosystems) or SYBR green. The cycling conditions were 50°C for 2 min and 95°C for 10 min, followed by 40 cycles at 95°C for 15 s and 60°C for 1 min.

### Challenge with VHSV

Forty (first immunisation experiment) or 60 (second immunisation experiment) days after DNA immunisation, all the remaining trout in each tank (15-20 trout per group) were challenged by bath-immersion with live VHSV (3x10^6^ TCID_50_ /ml). Mortality was recorded daily for 30 days and the relative percentage survival (RPS) was calculated by the formula: RPS = [1-(% mortality of immunized trout/ % mortality of control trout)] X 100.

### Estimation of anti-VHSV-gpG specific antibodies

The presence of specific antibodies against gpG_VHSV_ in trout sera was determined at days 30 post-vaccination by enzyme-linked immunosorbant assay (ELISA) using a recombinant fragment of VHSV-gpG (aa 56-110), the so called frag 11 [23], as an antigen in the solid-phase. Pools of sera (serum from 4 trout) from trout injected with PBS, or with the different plasmid constructs, were analysed and ELISA assays were carried out as previously described [12] using the monoclonal antibody to trout IgM, 1G7 [23]. As positive control, serum from VHSV_0771_ infection survival trout was used.

### Serum neutralising activity

The presence of neutralising antibodies was determined at days 30 post-vaccination. Briefly, 10 µl of serial 5-fold dilutions of trout sera pooled in reduced-serum culture medium (Opti-Mem, Invitrogen), beginning with a dilution ratio of 1:10 serial dilutions were incubated at 37 °C for 30 min to eliminate the complement present in the samples. Then, they were mixed with 1.5 µl of trout complement (fresh sera pooled form 5 healthy trout) in wells of 96-well plates (30 min at 14 °C). Fifty µl of a VHSV_0771_ dilution adjusted to 1x10^4^ f.f.u. /ml were added to the EPC monolayers and the plates incubated for 2h at 14 °C. After incubation, each serum-complement-virus mixture was added to EPC monolayers, grown in 96 well plates, in a final volume of 60 µl per well. Two hour post infection at 14 °C, infected cells were washed, 100 μl/ well of fresh cell culture medium (RPMI Dutch modified) supplemented with 2% FCS and plates further incubated for 24h at 14 °C. To evaluate VHSV_0771_ infectivity, the EPC cell monolayers were fixed for 10 min in cold methanol and air-dried. Monoclonal antibody (MAb) 2C9 directed towards the N protein of VHSV_07.71_ diluted 1000-fold in dilution buffer (0.24 mM merthiolate, 5 g/l Tween 20, 50 mg/l of phenol red in PBS, pH 6.8) were added to the wells (100 μl/well) and incubated for 1 h at room temperature. Peroxidase-labelled rabbit anti-IgG mouse antibody (100 µl) (Nordic, Tilburg, The Netherlands) were added per well, and incubation was continued for 30 min. After three washings by immersion in distilled water, 50 µl of 1 mg/ml per well of diaminobenzidine (DAB) (Sigma) in PBS containing H_2_O_2_ were added [24] and the reaction allowed to proceed until brown foci were detected with an inverted microscope (Nikon Eclipse TE2000-U, Nikon Instruments Inc., NY, USA). Brown foci of DAB stained cells (VHSV-infected cell foci) were counted with an inverted microscope with a 10× ocular eye grid. The results were expressed as inhibition of VHSV infectivity by the following formula, 100-(number of DAB positive foci in cultures inoculated with trout serum, complement and VHSV/ number of DAB positive foci in cultures inoculated with trout serum and VHSV in the absence of complement x 100).

### Statistical analysis

Data were analysed using a two tailed t-test (Graph Pad Prism 5 software) to determine the differences between non-immunised and immunised fish. Statistical differences were considered significant when p < 0.05. To determine the differences between groups in the gene expression assays, statistical comparisons were made using ANOVA followed by a post hoc test (Tukey’s). Statistical differences were considered significant when p < 0.01

## Results

### Characterisation of the VHSV-gpG protein forms expressed by stably transfected EPC cell lines

To avoid the variability related to transgene expression levels that is intrinsic to cell transfection assays, EPC cell lines enriched in cells stably-expressing the different VHSV-gpG forms (EPC-gpG_1-507_, EPC-gpG_1-462_ and EPC-gpG_LmPle20-462_) were generated and the expression of the modified VHSV-gpG proteins, at both transcriptional and protein levels, was analysed in each cell line. In addition, the expression levels of the VHSV-gpG forms in the stably-transfected cells were compared to those present in VHSV-infected EPC cells.

**Figure 2:**
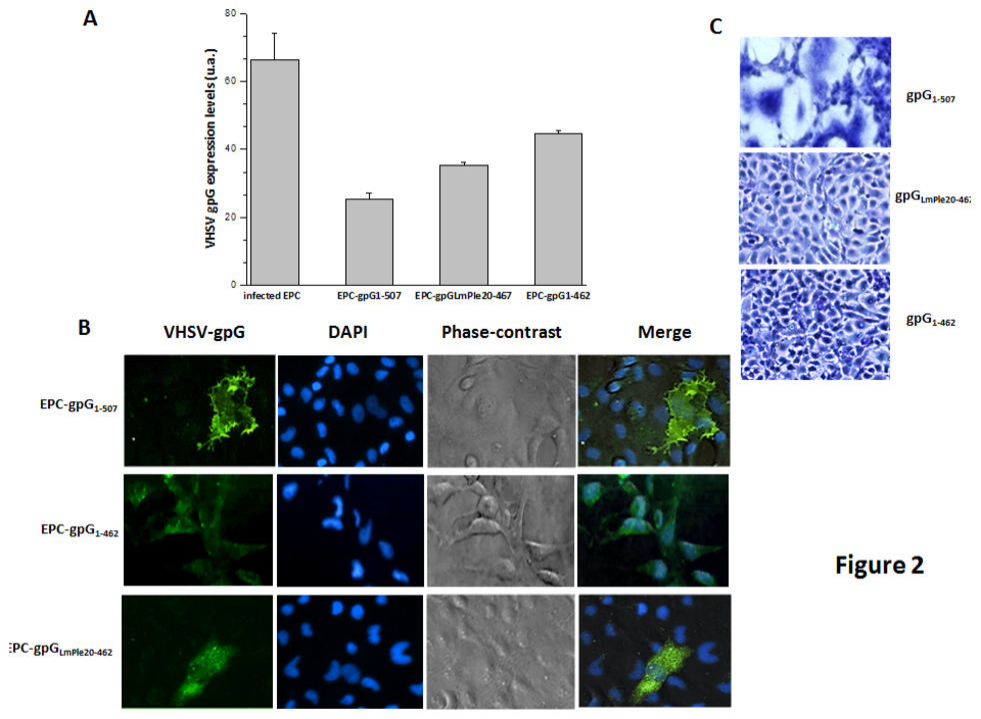
Characterisation of the VHSV-gpG protein forms expressed by the stably transfected EPC cell lines. Fresh EPC cell monolayers were co-transfected with each of pAE6-G_1-507_, pAE6-G_1-462_ and pAE6-G_LmPle20-462_, and pAE6-pac (puromycin resistance gene). After an incubation period with puromycin, the remaining cells were screened for the expression of the different constructs by qPCR (A) and immunofluorescence (B). A, Data shows the transcript levels of each VHSV-gpG construct as well as those of VHSV-infected EPC cells. B, Cells were stained using a cocktail of anti-gpG MAbs and fluorescein isothiocyanate (FITC)-labeled anti-mouse IgG Ab. EPC-gpG_1-507_ monolayers were fixed with citofix and the EPC-gpG_LmPle20-462_ and EPC-gpG_1-462_ monolayers permeabilised with 0,02% of PBS-Triton X.(C)To evaluate the functionality of the VHSV-gpG expressed by the transfected cells, a low pH-dependent fusion assay was performed. Cells were fixed with cold methanol, dried and stained with Giemsa (5 mg/ml in PBS).

Transcripts corresponding to the three different VHSV-gpG forms could be detected by RT-qPCR in their corresponding stably-transfected EPC cell lines indicating the successful transcription of the transgenes (Figure 2A). Although no important differences among the expression levels of the three VHSV-gpG forms were found, the most abundant transcripts were those corresponding to the soluble form (gpG_1-462_, ≥40-fold higher than those of the endogenous control gen). Interestingly, the lowest expression level was observed for the wild type form (gpG_1-507_) (Figure 2A). Finally, the highest levels of VHSV-gpG transcripts were detected in EPC cells infected with VHSV (approximately 60-fold higher than those of the endogenous gen) (Figure 2A).

### Cell expression patterns of the VHSV-gpG protein forms in stably transfected EPC cell lines

Having established the expression of the different VHSV-gpG forms, the expression pattern of the proteins in the stably-transfected cells was assessed by immunofluorescence assays. The wild type form of the protein (gpG_1-507_) was localised in the membrane of the cells (Figure 2B, upper panel). In contrast, neither the soluble nor the secreted soluble forms were found on cell membranes. Nevertheless, these two forms were detected in the cytoplasm of the cells, showing a very different expression pattern from that of the wild type form (Figure 2B, middle and lower panels). In the case of the secreted soluble form (gpG_LmPle20-462_) nearly all of the cells exhibited a non-uniform distribution of cytoplasmic granules, (Figure 2B, lower panel) resembling secretory vesicles. These cytoplasmic granules were not seen in the stably cells expressing the soluble form of VHSV-gpG (gpG_1-467_) where the fluorescence was uniformly distributed throughout the cytoplasm (Figure 2B, middle panel).

Next to evaluate the functionality of the constructs being expressed in the stably-transfected EPC cell lines, a low-pH dependent-fusion assay was performed. Syncytia could only be observed in the cell cultures expressing the membrane-anchored form, gpG_1-507_ (Figure 2C, upper panel), which after undergoing a low-pH dependent conformational change is able to reach the membranes of neighbour cells as previously reported [25].

Finally, the presence of the secreted soluble form of the VHSV-gpG in the culture supernatant of stably transfected EPC cell line (gpG_LmPle20-462_) was successfully assessed by HPLC (Figure S1).

### DNA immunisation and VHSV challenge experiments

**Figure 3:**
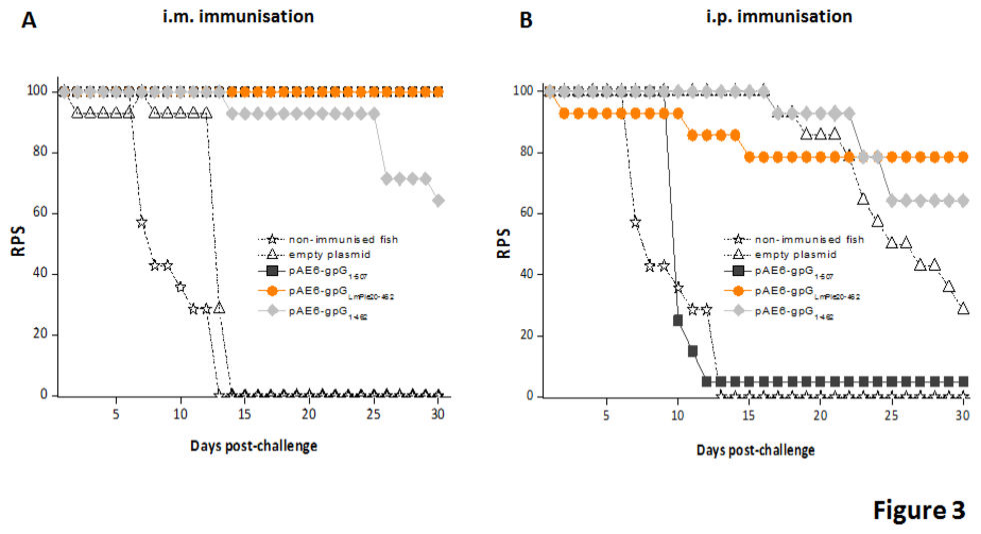
Protection against VHSV challenge conferred after DNA immunisation. Fish were immunised with each of the plasmid constructs intramuscularly (A) or intraperitonearly (B) in separated tanks. The challenge was carried out at 40 days after DNA immunisation, and mortality recorded for 30 days. Values are shown as Relative Percentage of Survival (RPS).

Immunized and non-immunised fish were challenged by bath-immersion 40 days post-immunisation with 3x10^6^ TCID_50_ VHSV-07.71/ml. Figure 3 shows the relative percentage of survival (RPS) from groups immunized intramuscularly, i.m. (A) or intraperitonearlly, i.p. (B), with each of the plasmid vaccine constructs (Table 2).

Significant protection was observed with a value of RPS from 80 to 100% in fish immunised with the plasmid encoding the secreted soluble form of VHSV-gpG (pAE6- gpG_LmPle20-462_) regardless of the route of immunization used (100% by i.m. and 80% by i.p. injection) (Figure 3A and B, orange circles). In contrast, the construction encoding the wild type form (pAE6-gpG_1-507_, grey squares) only conferred 100% protection when fish were immunised by i.m injection. Moreover, protection levels observed in i.m. or i.p.-immunised fish with the gpG soluble form (pAE6-gpG_1-462_, light grey diamonds) were low (64%).

As expected, non-immunised control fish had RPS values of 0% (Figure 3A and B, open stars). On the other hand, empty vector showed irrelevant protection on i.m. (Figure 3A, open triangles) but 28% on i.p. immunization route (Figure 3B, open triangles). Every dead fish presented extensive haemorrhages in the abdominal cavity, a typical symptom of VHSV infection (data not shown).

### Presence of specific antibodies and neutralisation activity in immunised trout sera with plasmids pAE6-G_LmPle20-462_ and pAE6-gpG_1-507_

Blood samples were collected 30 days after the immunization to analyse whether or not the protection rates conferred by the plasmids pAE6-gpG_LmPle20-462_ and pAE6- gpG_1-507_ for each of the immunisation routes correlated with the neutralising activity of specific antibodies anti-VHSV-gpG present in the sera of immunised fish. As control, a pool of sera from VHSV resistant fish (fish surviving VHSV infection) was used.

**Figure 4:**
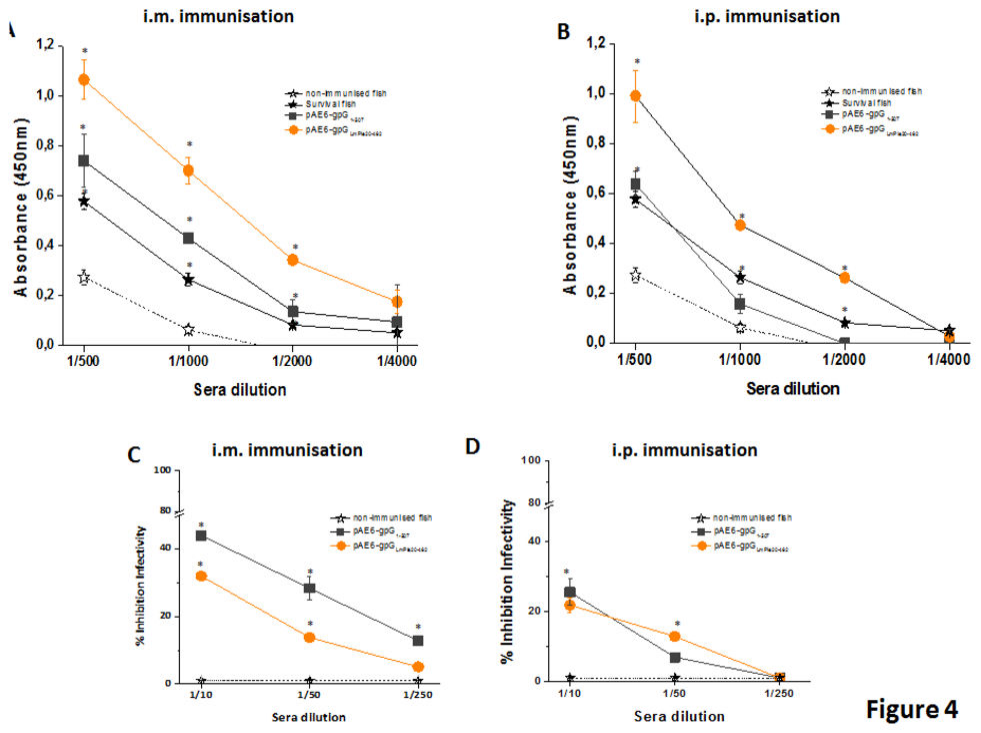
Specific IgMs anti-VHSV-gpG (A and B) and neutralizing activity in sera from immunised trout (C and D). The presence of specific antibodies against VHSV-gpG in trout sera was determined 30 days post-vaccination by an enzyme-linked immunosorbant assay (ELISA). Sera obtained from VHSV-survivor trout was used as a positive control. Absorbance readings were measured at 450 nm. Results represent the average values and standard deviations of sera from four fish per group, each assay in triplicate, from two different experiments where (A), is sera from fish immunised i.m. and (B), from fish immunised i.p. Neutralising assay from sera from fish immunised i.m. (C) or i.p.(D); VHSV_07.71_ was incubated with increased dilutions of sera from immunised or control trout as described in materials and methods. EPC cell monolayers were then infected with VHSV for 2h at 14 °C. Twenty four hours post-infection, the number of focus of VHSV_07.71_ were estimated as described in the material and methods section. Results were expressed as inhibition percentage relative to cells infected with untreated virus as calculated by the formula (100 − (number of DAB positive foci in EPC cultures treated with trout serum, complement and VHSV_07.71_ /number of DAB positive foci in EPC cultures treated with trout serum and VHSV_07.71_ in the absence of complement) × 100). Symbols represent the average values and standard deviations (error bars) of two independent experiments each performed in duplicate. The sera dilution marked with “*” showed statistically significant differences with control fish at p< 0.05 according to the t-test.

Thirty days after immunisation, all of the sera samples from fish immunised with either pAE6-gpG_LmPle20-462_ (orange circles) or pAE6-gpG_1-507_ (grey squares) by i.m. as well as i.p. injection contained high titters of gpG-specific antibodies (Figure 4A and B); additionally, no specific antibodies were detected in the sera from control fish (open stars). Titers from sera of immunised fish were higher or similar to those of sera from trout surviving an infection with VHSV (black stars, Figure 4A y B). The highest specific antibody levels were found in sera from trout immunised by i.m. or i.p injection with the secreted soluble form of VHSV-gpG, pAE6-gpG_LmPle20-462_. Regardless of the plasmid used, the titers of specific antibodies were higher when the DNA vaccines were administered by i.m. injection (Figure 4A and B).

The neutralisation capacity of the antibodies present in the sera of immunised fish was also tested using an immunostaining focus assay previously developed for the immunodetection of viral haemorrhagic septicaemia virus strain 07.71 [12]. None of the tested sera showed effective neutralising activity since the maximal decrease of VHSV infectivity after virus neutralisation was ≤55% in the fish immunized by injection with the wild type form of VHSV (pAE6-gpG_1-507_) but only at the lower sera dilution (1/10) (Figure 4B and C). Taken together these results suggest that virus neutralisation by sera specific antibodies is not the underlying cause of protection conferred by the vaccines.

### Differential gene expression patterns induced by pAE6-gpG_LmPle20-462_ and pAE6-gpG_1-507_ after i.p injection.

Prior to carrying out the gene expression analysis we checked the in vivo the expression of the different forms of VHSV-gpG since the in vitro conditions governing the expression of the proteins encoded by the plasmids might be different from those in vivo. For that, fish were injected with the plasmids pAE6-gpG1-507, pAE6-gpGLmPle20-462 or pAE6-gpG1-462 by i.m. or i.p injection and the expression of VHSV-gpG analysed at day three post-injection. As in the in vitro assays, transcripts corresponding to the three different VHSV-gpG forms could be detected by RT-qPCR in muscle (i.m. injected fish) and intraperitoneal cavity (i.p. injected fish) cells, indicating the successful transcription of the transgenes (data not shown).

Since no differences were found among the RNA expression levels of the three forms of VHSV-gpG *in vivo* (data no shown), to investigate what causes the differences among protection rates after VHSV lethal challenge conferred by i.p. injection of the plasmids pAE6-gpG_LmPle20-462_ and pAE6-gpG_1-507_, the transcript levels of several immune-related genes were estimated by RT-qPCR (Table 1) in cells extracted from the intraperitoneal cavity of immunised and non-immunised fish at day 3 post immunisation. Overall, fish injected with the empty plasmid (pAE6) in most cases increased the levels of expression of immune-related genes when compared to those of fish injected with PBS, in agreement with previous studies [12]. For that reason, transcript levels of fish injected with the empty plasmid were used as calibrator to evaluate the gene expression, in other words, pAE6-injected fish served as control fish for transcript level determination.

**Figure 5:**
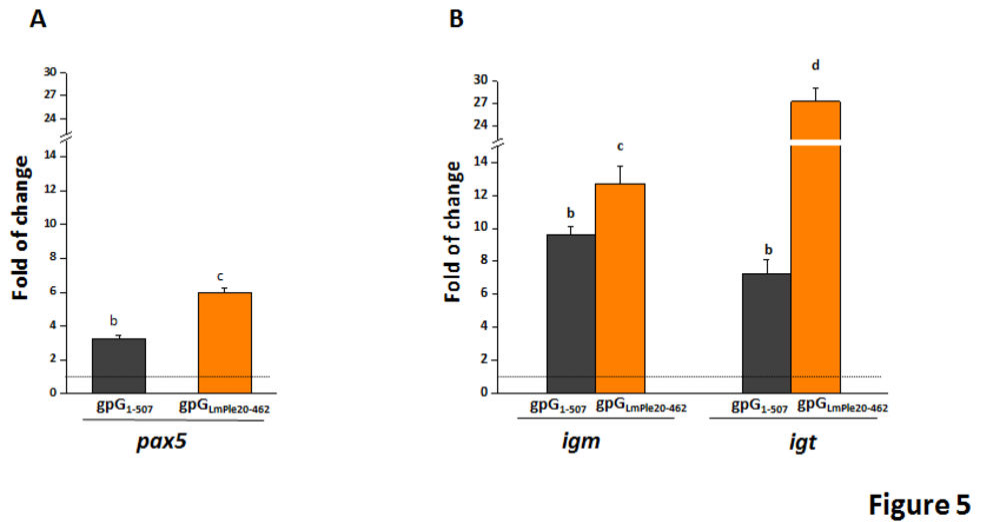
Transcripts levels of *pax5*, *igm* and *igt* in peritoneal cells 3 days after immunisation. Levels of *pax5* (A), *igm* and *igt* (B) transcript levels estimated by RT-qPCR in fish i.p. injected with 2 µg of either pAE6-gpG_1-507_ (black bars) or pAE6-gpG_LmPle20-462_ (orange bars). Bars represent the mean fold changes and SD of pools of 3 fish per group. Fold increases were calculated relative to the level of non-immunised fish (injected with PBS). Different letters denote statistically significant differences between the groups according to a Tukey’s test.

At the primary site of antigen delivery (intraperitoneal cavity), the presence of B cells was evaluated by means of the analysis of the expression levels of the B-cell-specific transcription factor *pax5* which, within the B cell linage, is expressed from the pre-B cell through mature B cell stages [26]. Overexpression of *pax5* was observed at day 3 after immunisation in the fish immunised with both gpG-containing plasmids (~6- and 3-fold increase for pAE6-gpG_LmPle20-462_ and pAE6-gpG_1-507_, respectively) (Figure 5A) suggesting that the immunisation with the gpG of VHSV induces a specific B cell infiltration to the injection site at early times post-immunisation. In fish immunised with the construction encoding the gpG secreted soluble form (pAE6-gpG_LmPle20-462_) the potential B cells infiltrated in the intaperitoneal cavity, according to *pax5* expression levels, were almost 2-fold higher than in the fish immunised with the plasmid encoding the wild type form (pAE6-gpG_1-507_).

To further characterise the migrating B cell population, transcript levels of membrane *igm* and *igt* were determined. Gene transcript levels obtained showed increased presence of B cell-membrane *igm* (~12- and 9-fold for pAE6-gpG_LmPle20-462_ and pAE6-gpG_1-507_, respectively) and *igt* (~27- and 7-fold for pAE6-gpG_LmPle20-462_ and pAE6-gpG_1-507_, respectively) regardless of the plasmid used (Figure 5B). However, while membrane *igt* expressing cells were the most abundant B cell population in pAE6-gpGLmPle_20-462_-immunised fish, membrane *igm* expressing cells were the predominant cell type in pAE6-gpG_1-507_-immunised fish. In fact, the *igt* expression ratio between fish immunised with the secreted soluble and wild type construction (gpG_LmPle20-462 /_ gpG_1-507_) was 3.7, suggesting that the increased presence of membrane *igt* expressing cells might participate in the orchestration of the protective immune response induced by the construction encoding the secreted soluble form of VHSV-gpG (Figure 5B).

**Figure 6:**
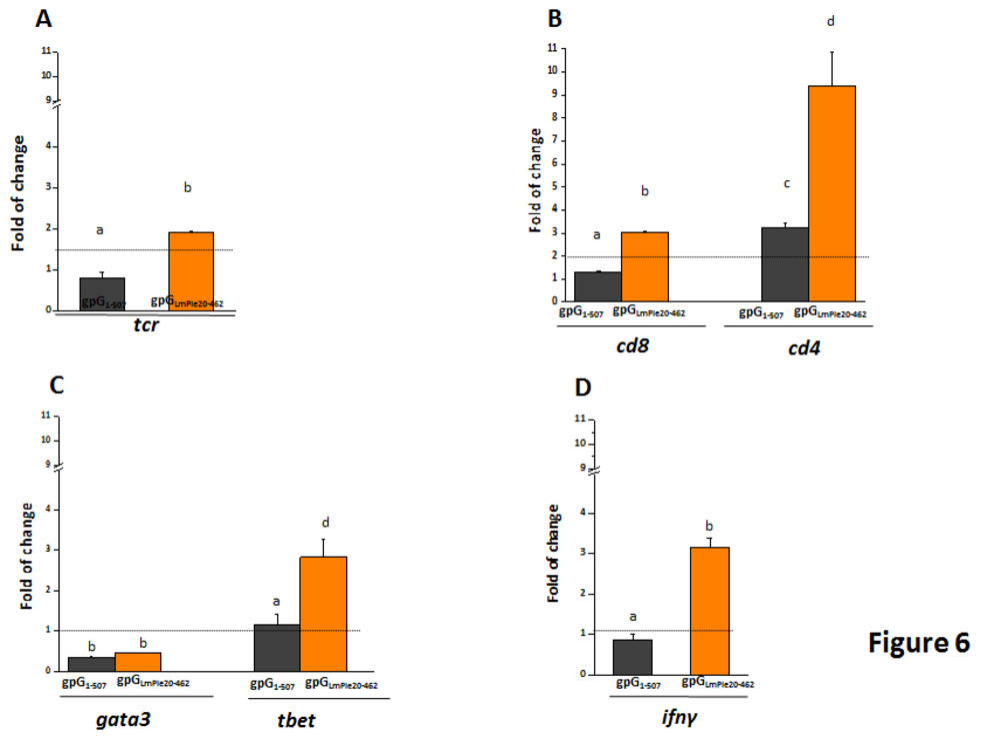
Transcripts levels of genes related to differentitation of T lymphocytes in peritoneum cells at 3 days after immunisation. Levels of *tcr* (A), *cd4* and *cd8* (B), *gata3* and *tbet* (C) and *ifnγ* (D) gene expression estimated by RT-qPCR in fish i.p. injected with 2 µg of pAE6-gpG_1-507_ (black bars) or pAE6-gpG_LmPle20-462_ (orange bars). Bars represent the mean fold changes and SD of pools of 3 fish per group. Fold increases were calculated relative to the level of non-immunised fish (injected with PBS). The differences among groups were analysed by ANOVA with a post hoc test (Tukey’s). Different letters denote degrees of statistical significance in the differences between the groups according to a Tukey’s test. The groups marked with “a” did not show statistically significant differences from control fish.

The presence of T cells in the fish peritoneal cavity was also evaluated by analysing the transcription expression levels of the T cell receptor (tcr). The *tcr* expression levels found in pAE6-gpG_1-507_ immunised fish were similar to that of control fish. In contrast, *tcr* expression levels were 2,4-fold higher in pAE6-gpGLmPle_20-462_-immunised fish, indicating that the secreted soluble form of VHSV-gpG might be more efficient in inducing the recruitment of T cells (Figure 6A). Gene expression of the T cell markers *cd8* (T cytotoxic cells) and *cd4* (T helper cells) were also estimated to characterise T cells and VHSV-gpG-specific recruitment of *cd8* cells was observed only in pAE6-gpG_LmPle20-462_-immunised fish (Figure 6B).

T helpers, on the other hand, were the predominant T cell type present in the peritoneal cavity of immunised fish as shown by *cd4* gene expression levels (9.4- and 3.2 fold for pAE6-gpGLmPle_20-462_ and pAE6-gpG_1-507_, respectively) (Figure 6B). In immunised fish, decreased transcript levels of *gata3* (Figure 6C), a specific transcription factor (TF) of Th2 cells [27], suggests that these fish mount a Th1-biased immune cell response. Curiously, when the expression levels of *t-bet*, a Th1-specific T box transcription factor that controls the expression of the hallmark Th1 cytokine IFNγ was analysed, only the fish immunised with the secreted soluble form showed significant differences compared to control fish. As expected, *t-bet* expression correlated with IFNγ expression (3,1- and 0,8-fold for pAE6-gpGLmPle_20-462_ and pAE6-gpG_1-507_, respectively) (Figure 6D). All together, these results suggest that a true polarised Th1 immune response is only induced *in vivo* by the secreted soluble form of VHSV.

### Effect of Montanide, a commercial fish vaccine adjuvant, in the protective response induced by the i.p immunisation with the pAE6-gpG_LmPle20-462_ plasmid.

Since only the secreted soluble form of VHSV-gpG conferred protection by both i.m. and i.p. routes, the oil-adjuvanted DNA vaccine assays (Table 2) were carried out with this plasmid pAE6-gpG_LmPle20-462_. Briefly, pAE6-gpG_LmPle20-462,_ pAE6 (empty plasmid) or PBS were mixed with the non-mineral oil adjuvant Montanide. All groups of i.p.-injected fish were challenged with VHSV 60 days after immunisation. RPS values with or without the adjuvant were similar (75%) (data not shown). No lesions or delayed growth fish rates were observed during this study.

**Figure 7:**
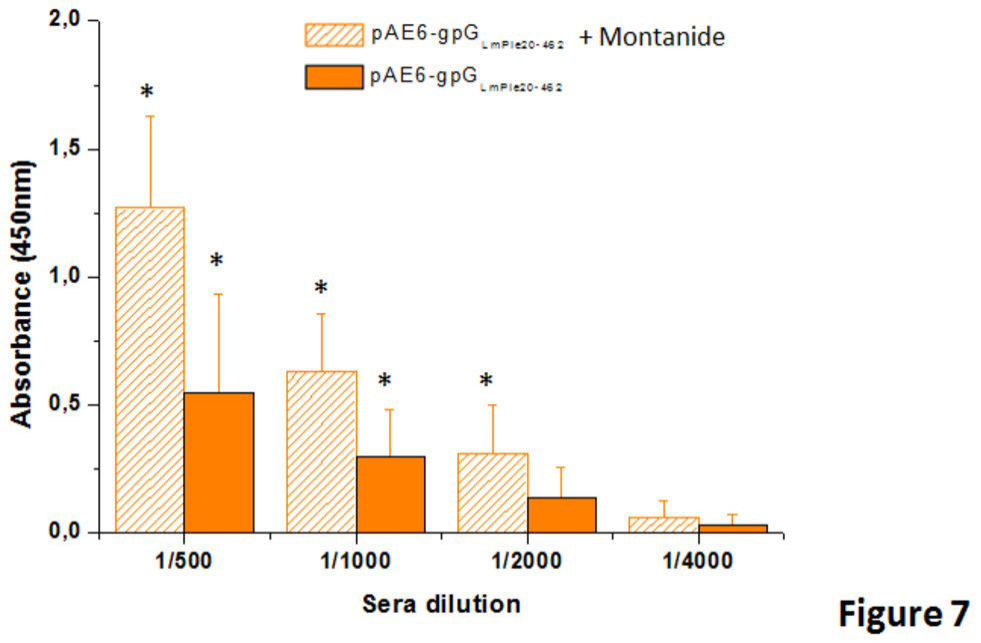
Specific IgMs anti-VHSV-gpG in sera of trout vaccinated with pAE6-G_LmPle20-462_ or pAE6-G_LmPle20-462_ and Montanide. The presence of anti-VHSV-gpG antibodies in the samples vaccinated with the pAE6-gpG_LmPle20-462_ or pAE6-gpG_LmPle20-462_ and Montanide in trout sera were determined at 30 days post-vaccination by enzyme-linked immunosorbant assay (ELISA), as described in the materials and methods section. Absorbance readings were measured at 450 nm. Bars represent the average values and standard deviations of sera from four fish per group, each assay in triplicate. pAE6-gpG_LmPle20-462_ (orange bars), pAE6-gpG_LmPle20-462_ and Montanide (hatched bars). The sera dilution marked with “*” showed statistically significant differences from control fish at p< 0.05 according to a t-test.

The presence of specific antibodies in the sera of fish immunised with either pAE6-gpG_LmPle20-462_ plasmid or pAE6-gpG_LmPle20-462_ plasmid +Montanide were also determined 30 days post-vaccination. Increased specific antibodies in sera from fish immunised with pAE6-G_LmPle20-462_ in the presence of Montanide were observed (Figure 7).

## Discussion

Although a large amount of knowledge on DNA vaccines has been accumulated over the past 15 years, the mechanism by which the different routes of vaccine delivery prime the immune system have not been fully investigated. Therefore, it is not possible to choose the most convenient administration route in each situation, strongly limiting the versatility of DNA vaccines.

In this work, using as a model the DNA vaccine based on the glycoprotein gene of VHSV, we have modified the subcellular location of the plasmid-encoded antigen expression in *in vivo* transfected cells to study and look for possible solutions to the problems related to route-dependent protection of this vaccine. To that end and based on a plasmid encoding the wild type form of VHSV-gpG (membrane anchored protein) [12], we have designed plasmids encoding a soluble (pAE6-gpG_1-462_) or a secreted soluble (pAE6-gpG_LmPle20-462_) form of the viral protein. Soluble forms of viral surface glycoproteins of other viruses such as rabies virus or human cytomegalovirus [28,29] have been also used with the aim of increasing their immunogenicity for vaccine purposes. However, replacing the signal peptide of a viral glycoprotein with the signal peptide of a naturally secreted antimicrobial peptide, the pleurocidin, is a novel strategy that has not been tested yet. The pleurocidin signal peptide was chosen because of the abundance of this peptide in the skin mucous secretions of fish [9], which indicates that it possesses a very efficient secretion mechanism in fish.

After determining the correct expression and subcellular location of the different forms of VHSV *in vitro*, fish were immunised by i.m. and i.p. rout with each of the three forms of VHSV-gpG (membrane anchored, soluble and secreted soluble protein) and protection rates after VHSV lethal challenge recorded. The plasmid encoding the soluble secreted form (pAE6-gpG_LmPle20-462_) was the only one that conferred protection by both the i.m. and i.p. routes, being this the first description of a fish viral DNA vaccine that succeeds in conferring protection when administered by intraperitoneal route. Moreover, these protection rates correlated with the levels of specific antibodies against VHSV-gpG since antibodies titers were in all cases higher in the fish immunised with the pAE6-gpG_LmPle20-462_ regardless of the plasmid administration route used. On the other hand, the differences in the antibody response elicited by the different forms of VHSV-gpG were not due to different protein expression levels produced since the three forms of VHSV-gpG were made with an identical plasmid backbone and produced similar RNA levels *in vivo*. Therefore, as reported previously for others proteins [30,31], the secreted form of an antigen induces the highest antibodies responses after DNA immunization. However, since none of the sera containing specific antibodies against VHSV-gpG showed effective neutralising activity, the results suggest that virus neutralisation by sera specific antibodies is not the underlying cause of protection conferred by the vaccines.

A plausible explanation of why the secreted VHSV-gpG induced higher antibody responses than the other forms is that the differences observed were due to antigen availability for the priming of B cells. In addition to the potential role played by the B cells, and as some studies have demonstrated [32], the differential antigen localization could be influencing a preferential induction and proliferation of different Th cell subsets. To investigate this, the presence of B and T cells in the fish peritoneal cavity at 3 days post-immunisation was analysed by evaluating the transcription levels of specific B and T cell markers. Overall, the results showed increased infiltration of both B cell and T cells in response to the secreted soluble form of VHSV-gpG at early time post-immunisation.

Regarding B cells, while membrane *igm* expressing cells were the predominant B cell subpopulation in pAE6-gpG_1-507_-immunised fish, membrane *igt* expressing cells were the most abundant in pAE6-gpG_LmPle20-462_-immunised fish. Similarly, high titter specific antibodies and high expression levels of the *igt* gene were previously detected in the spleen of fish intramuscularly immunized with pAE6-gpG_1-507_ plasmid [12]. Taken together these results suggest a direct implication of B cells, and particularly of a subset of B cells expressing the membrane form of IgT, in the orchestration of the protective immune response at early times post DNA immunisation. To note that fish B cells have *in vivo* phagocytic activities [33] which suggest an important participation of these cells in the crosstalk that link innate and adaptive immune processes. Therefore, fish B cells probably play a major role after DNA vaccination compared to their mammalians counterparts. On the other hand, a true polarised Th1 immune response was only induced *in vivo* by the secreted soluble form of VHSV-gpG.

Since the most commonly used adjuvants in fish intraperitoneal vaccines are oils solutions, we evaluated the effect of an oil adjuvant (Montanide ISA 763 AVG) on the response induced by the intraperitoneal immunisation of fish with the plasmid encoding the secreted soluble for of VHSV-gpG (pAE6-gpG_LmPle20-462_). Results showed that the protection rates conferred by the DNA vaccine were similar regardless of the presence of Montanide. Since oil adjuvants are known to enhance the humoral response [34], antibody titers displayed by the trout i.p. immunised with pAE6-gpG_LmPle20-462_ together the oil adjuvant were higher than those obtained with the plasmid pAE6-gpG_LmPle20-462_ alone.

In light of these results, DNA vaccines for salmonid fish that only conferred protection by i.m. injection so far, could now be administered in combination with other fish multivalent vaccines that are routinely administered to the fish by i.p. injection. This should increase the versatility of DNA vaccines against fish rhabdoviruses and will be of definitive interest for the aquaculture industry.

## Supporting Information

Figure S1 **Chromatographic profile of supernatants from the different stably transfected EPC cell lines.** Supernatants were harvested 5 days after cell seeding in 25 cm^2^ flasks, eluted through a C18 column, at 0.5ml/min, that separate the molecules according to their hydrophobicity, using a linear gradient from solution A [water with 0.12% trifluoroacetic acid (TFA)] to 60% solution B (acetonitrile with 0.10% TFA) in 60 min. Arrows, peacks corresponding to VHSV-gpG.
(TIF)
