# Supplementary figures and images for: Increasing Versatility of the DNA Vaccines through Modification of the Subcellular Location of Plasmid-Encoded Antigen Expression in the In Vivo Transfected Cells
Source: PLoS One. 2013 Oct 9;8(10):e77426. doi: 10.1371/journal.pone.0077426 (PMC3794048; doi:10.1371/journal.pone.0077426)

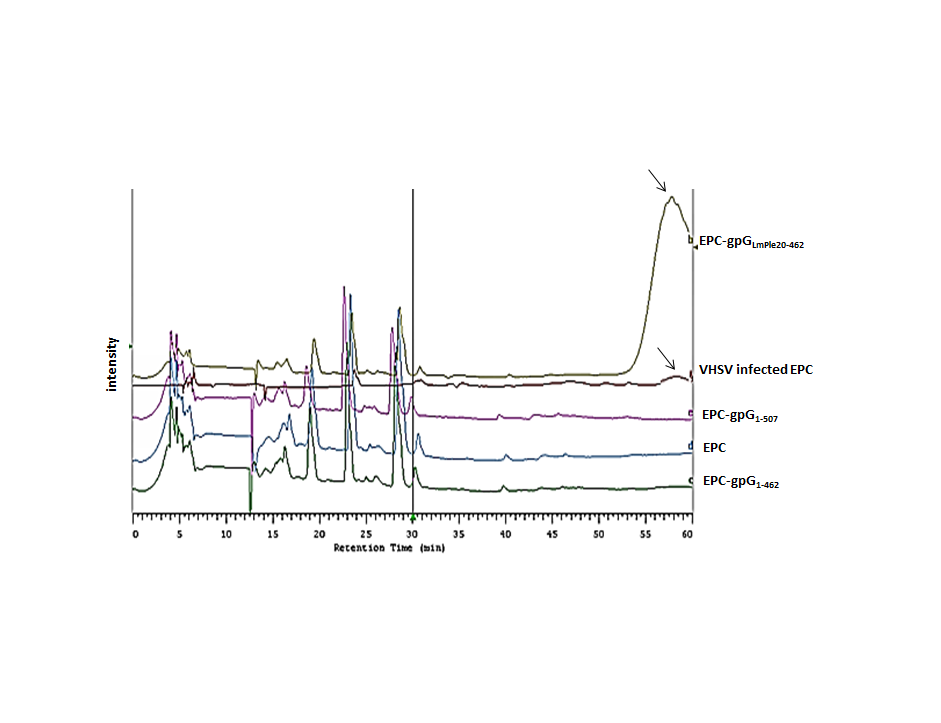

Supplement: Figure S1 — Chromatographic profile of supernatants from the different stably transfected EPC cell lines. Supernatants were harvested 5 days after cell seeding in 25 cm2 flasks, eluted through a C18 column, at 0.5ml/min, that separate the molecules according to their hydrophobicity, using a linear gradient from solution A [water with 0.12% trifluoroacetic acid (TFA)] to 60% solution B (acetonitrile with 0.10% TFA) in 60 min. Arrows, peacks corresponding to VHSV-gpG. (TIF) [file pone.0077426.s001.tif]
